# Pre-prosthetic Physiotherapy Rehabilitation in Post-operative Transtibial Amputation in a Patient With Congenital Talipes Equinovarus

**DOI:** 10.7759/cureus.29724

**Published:** 2022-09-29

**Authors:** Aditi Joshi, Rupali Thorat, Priyanka A Telang

**Affiliations:** 1 Physiotherapy, Ravi Nair Physiotherapy College, Datta Meghe Institute of Medical Sciences (DU), Wardha, IND; 2 Community Health, Ravi Nair Physiotherapy College, Datta Meghe Institute of Medical Sciences (DU), Wardha, IND

**Keywords:** gait training, physiotherapy rehabilitation, gangrene, wedge tarsectomy, congenital talipes equinovarus, amputation

## Abstract

Amputation is more common in men than women, a lot of studies suggest this. It is the complete or partial removal of an extremity through a surgical process and is said to be a life-saving procedure performed in various critical conditions. The main objective of amputating a limb or any part at a level is that it should be carried out in a way that will give a stump of optimum length to facilitate the prosthetic fitting at later stages. After amputation, the patients are usually trained with prostheses so that they can carry on with functional activities without any restrictions. One of the disorders seen in infants and children is congenital talipes equinovarus (CTEV). It is characterized by plantarflexion at the ankle joint, inversion at the subtalar joint, and adduction at the forefoot. There are various factors and causes associated with CTEV. The treatment should be done as early as possible, if delayed, it can lead to deformities in the joint. Here is a case of a 12-year-old male, who, a neglected case of bilateral clubfoot, now underwent wedge tarsectomy surgery for the left foot and below knee amputation of the right leg due to the formation of infectious gangrene. Post-surgery, the patient was referred to the physiotherapy department for further treatment and rehabilitation.

## Introduction

Amputation is defined as the removal of a limb through a part of the bone surgically. It is a life-saving treatment used in a variety of severe situations. The main goal of amputating a limb or any component at a particular level is to allow a stump length for optimal prosthetic fitting in the future. The transtibial amputation is one of the most common major limb amputations practiced all over the world. The long posterior flap technique is the most commonly advised procedure, as it helps in the proper fitting of prostheses in the future [1]. There are many infants now who are suffering from clubfoot, also known as congenital talipes equinovarus (CTEV), one of the most common congenital anomalies. The word talipes is derived from “talus” and “pes” and was applied to those walking on their neglected deformities wherein the talus rested on the ground as the foot (pes) [2]. It is characterized by plantar flexion at the ankle joint, inversion at the subtalar joint and adduction at the forefoot. It is observed that the inner border of the foot is raised and shortened with cavus, an exaggerated longitudinal arch and an outer border of the convex foot bear weight with a thickened skin area [3]. The finest therapy can be offered immediately after the child's birth, however, in impoverished and however, poverty, education, and limited resources in developing countries may lead to neglect [4]. Here, we present a case of a male teenager, who visited the hospital with a complaint of gangrene on their right foot, he had a history of bilateral CTEV. The gangrene was treated and performed a transtibial amputation of the right leg by the orthopedic surgeon, and later, a wedge tarsectomy was also conducted for the left foot. Since both lower limbs of the patient were operated on, his functional and independent activities were restricted, and he became dependent and weak. To recover and achieve activities of daily living, the patient was referred to the physiotherapy rehabilitation department and there, the therapists planned a rehabilitation program to attain functional activities and enhance the quality of life.

## Case presentation

A 12-year-old male was brought to the hospital with a history of injury to the left ankle and severe pain. History revealed that the patient had developed infection post-injury due to a lack of medical care provided early. Due to the infection, there was swelling all over the right foot, and the development of gangrene. The patient was planned for below knee amputation, i.e., transtibial amputation of the right leg to control the spread of pain, swelling, and infection. The patient was presented with bilateral CTEV, and for many years was walking on the lateral border of feet due to which callosities were formed. On investigation, the reason behind the neglect and late presentation was compromised economic status and less family support. A detailed physical examination was performed after gathering the basic patient information. There was no family history of CTEV. A thorough examination revealed idiopathic Bilateral CTEV with severely deformed feet. The patient walked bearing weight on the dorsal aspect of the lateral border of both feet which had large callosity with an underlying bursa. Left wedge tarsectomy surgery was planned. After the surgery, the patient was referred to the physiotherapy department for further management and rehabilitation.

Clinical findings

On observation, the patient was awake and oriented to time, place, and person. After taking the patient’s and family’s consent, the patient was examined thoroughly. He was ectomorphic inbuilt and inspected in the supine lying position. The patient had undergone right below knee amputation because of infectious gangrene. Since he was a case of neglected clubfoot, due to the presence of callosities, wedge tarsectomy surgery was performed on the left foot. On palpation, the local temperature was raised slightly around the stump. Tenderness was present with grade 2 (patient had pain and winces, on palpation), according to tenderness grading scale. Sensations on both legs were intact whereas, the strength of both the lower limbs was reduced. On the medical research council (MRC) scale of grading, muscle strength was 3/5 in both lower extremities. The range of motion of the lower limb is given in Table 1.

**Table 1:** Range of motion of lower limbs on first day of rehabilitation

Joint	Movements	Right (active ranges)	Right (passive ranges)	Left (active ranges)	Left (passive ranges)
Hip	Flexion	108	115	110	115
	Extension	20	25	18	25
	Abduction	25	30	20	30
	Adduction	25	30	25	30
	Internal rotation	30	30	25	30
	External rotation	28	35	30	35
Knee	Flexion	114	120	115	120
	Extension	0	0	0	0

Therapeutic interventions

**Figure 1:**
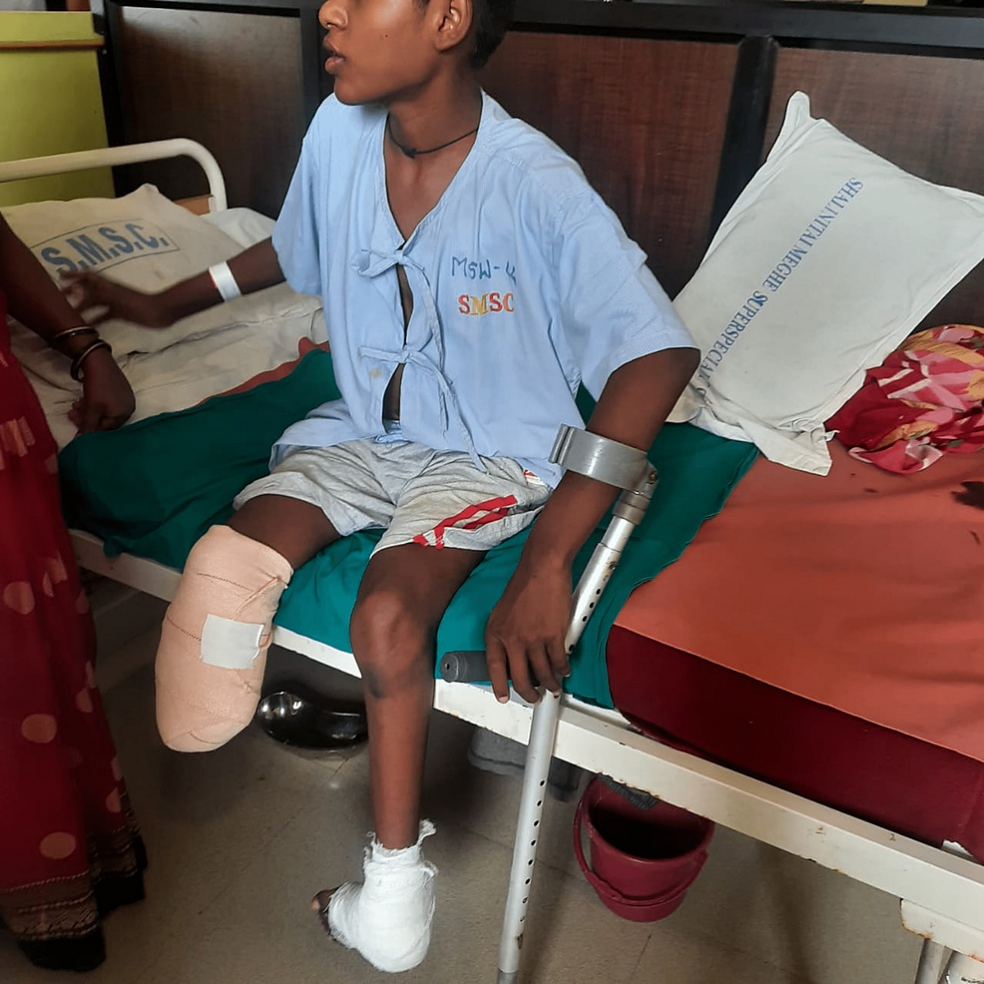
Active movements for limbs

**Figure 2:**
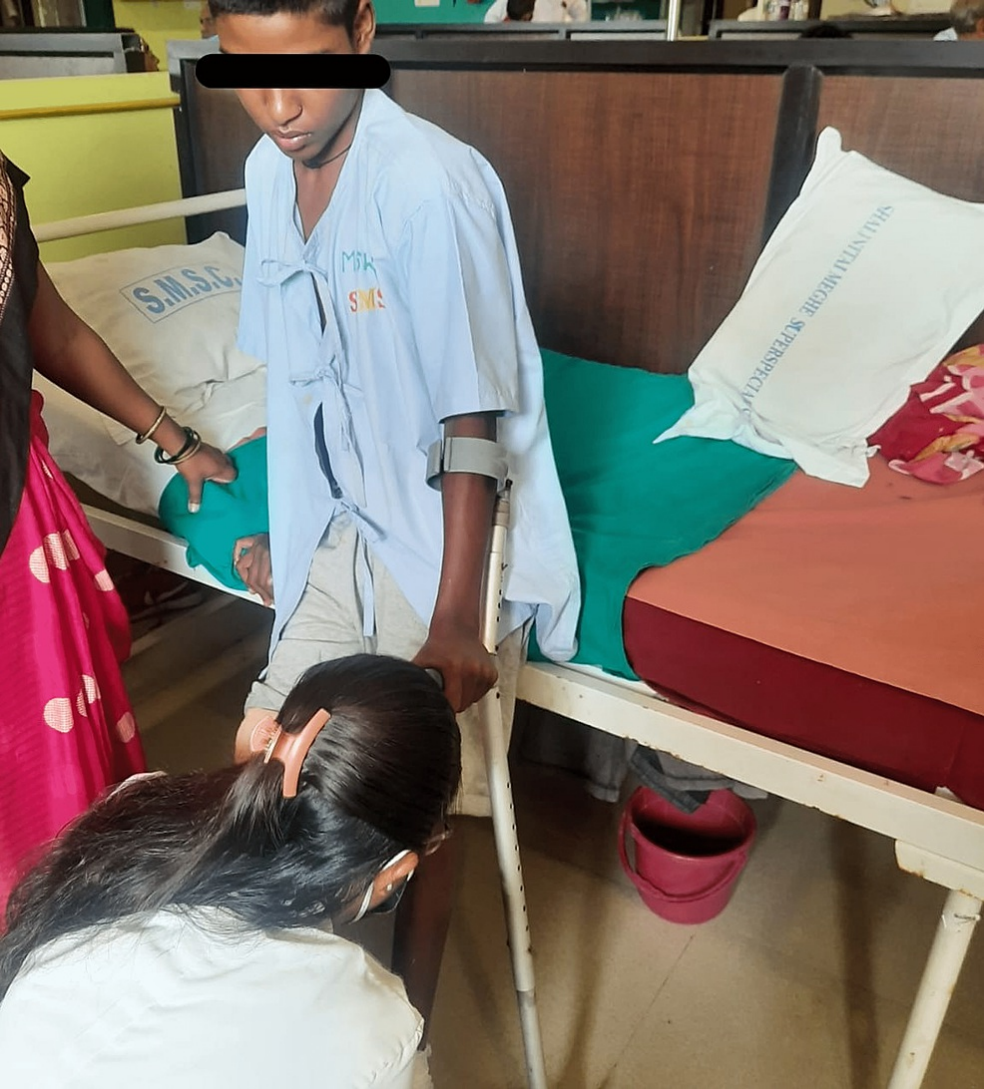
Gait training and correct foot placement

The surgery was performed, for right below knee amputation, to prevent the spread of infection. The second surgery was of wedge tarsectomy for CTEV of the left foot. The patient was reported to the physiotherapy department after surgery. After taking the patient’s and family’s consent and undergoing a thorough assessment, rehabilitation was planned for the patient. The primary goals were to increase muscle strength, improve range of motion and prevent stiffness and further deformities. After physiotherapy rehabilitation, the patient was suggested for a below knee prosthesis, which would help the patient to ambulate independently. The physiotherapy rehabilitation protocol is shown in detail in Table 2. In Figure 1, the patient is performing active range of motion exercises for the right knee joint. The therapist is supporting the foot while gait training (Figure 2). 

**Table 2:** Physiotherapy rehabilitation protocol

Sr no.	GOALS	INTERVENTION
1	Education to patient and his family.	The patient and family members were educated about the procedures taken place and further treatment and importance of physiotherapy rehabilitation. Gaining cooperation and consent from patient and his family.
2	Scar Management and care	Initially proper scar management was provided with proper dressing, the amputed limb was slightly elevated to prevent edema.
3	To improve bed mobility	Monitored bed transitions and bedside sitting. The patient was taught rolling, side lying to sitting, positions which help in preventing complications like bed sores. Proper positioning was taught to patient and care giver to prevent contracture and advised to change position in every few hours initially.
4	Active range of motion exercises of upper limb.	Range of motion exercises for all joints of both the upper limbs. Daily, 8-10 repetitions to each joint actively. This maintained the joint mobility.
5	Active exercises to lower limbs	Hip and knee active movements were encouraged for both right and left lower limbs to maintain the joint mobility and prevent deformities. Active knee extension was encouraged to amputed leg, passive stretching and positioning provided by elevation on bed. Toe movements of left leg were also taught.
6	Strength training	The patient was taught strengthening exercises for right knee extensors and hip flexors of the right amputed leg using soft ball to make session interesting for the child. Progressive resistive exercises were gradually given to Knee flexors and extensors and hip flexors, extensors, abductors, adductors of left leg post wedge tarsectomy. Sit to stand activity, free movements of limbs in standing were given to gain strength and attain balance. Strengthening to both upper limbs was given to maintain the strength in upper limbs.
7	Gait Training	The patient was taught to first maintain balance. Gait training was first started on parallel bar. Maintenance of foot in corrected position was taught by the physiotherapist by supporting the foot in corrected position. Use of walker was taught followed by use of elbow crutch and three- point gait was taught by visual feedback.
8	Home exercise program.	A proper home exercise program was taught to the patient and his care givers. Use of orthosis to maintain the corrected foot posture and prevent recurrence of the deformity. Education of stump cleaning and hygiene was given to patient and his parents. They were educated about benefits and importance of prosthesis at later stages of life.

Outcome measures

The manual muscle testing post rehabilitation on the MRC grading scale for lower limb - 4/5. The Amputee Mobility Predictor without prosthesis (AMPnoPRO) score was - 26/39. Table 3 shows the ranges of joints after rehabilitation.

**Table 3:** Range of motion post rehabilitation

Joint	Movements	Right (active ranges)	Right (passive ranges)	Left (active ranges)	Left (passive ranges)
Hip	Flexion	115	120	112	115
	Extension	25	30	20	25
	Abduction	28	35	30	35
	Adduction	30	37	38	42
	Internal rotation	30	35	30	35
	External rotation	32	38	35	40
Knee	Flexion	125	130	120	126
	Extension	0	0	0	0

## Discussion

Amputation is a life-saving procedure used in various critical situations. The transtibial amputation is one of the most common major limb amputations performed around the world [1]. Many infants are now born with clubfoot, also known as CTEV, which is one of the most common congenital anomalies [2]. CTEV, or clubfoot, is a structural abnormality that occurs early in pregnancy. Its prevalence at birth varies between and within low- and middle-income countries (LMICs), and this information is required to plan treatment programs. Improving and maintaining the range of motion of joints of lower limbs, strengthening the muscles, balance exercises, gait and functional activities of daily living with locomotion were the main objectives of physiotherapy rehabilitation for the benefit of the patient during the recovery stage postoperatively. However, due to surgery done for both lower limbs, the patient had difficulty in ambulation. The presence of cast post wedge tarsectomy restricted the ranges of the ankle, the movements possible were only for toes [5]. This report included training the strength of the major group of muscles, quadriceps, hamstrings, adductors, and abductors of both lower limbs so that the patient is able to perform activities of daily living independently [6]. Because each patient will opt not to wear the prosthesis at all times, he must be independent while not wearing it in ordinary life; consequently, strength training is essential [7]. Strach, in his research, stated that CTEV treatment begins as soon as possible after birth, with repeated manipulation and fixation by strong bandages that should be maintained for a long time to achieve over correction [3]. But in neglected CTEV the management aims to reduce, if not eliminate, all elements of the clubfoot deformity, resulting in a functional, pain-free, normal-looking plantigrade, mobile, callous-free, and normally shoeable foot. After the corrective surgery is performed in neglected CTEV cases, the patient is referred for physiotherapy treatment so as to progress and perform basic functional activities with ease and independence [2]. Alkjaer in his study said that when comparing gait kinematics and kinetics using a single-segment foot model, adults who received the early surgical intervention were similar to uninvolved individuals, stating that early surgery will lead to improved gait patterns [8]. In this report, the patient had raised concern about continuing exercises post discharge without professional monitoring or supervision for which he and his family were trained for proper home exercise programs and educated well [9,10].

## Conclusions

CTEV is found in every two in 1,000 live births. It usually results because of some abnormality or in cases of complicated pregnancies. Following the rehabilitation, the patient was able to perform all functional activities independently and showed significant improvement in joint range of motion, muscle strength, posture, and pain, preventing further complications in the future. This case report establishes a physiotherapy rehabilitation protocol that can be used for below knee amputation, preparing for prosthesis and gait training for clubfoot after corrective surgery. The process can be used in future cases to assess the beneficial outcomes to the patient in that geographic area.
